# Prevalence and risk factors of secondhand smoke (SHS) exposure among pregnant women in Mongolia

**DOI:** 10.1038/s41598-017-16643-4

**Published:** 2017-11-27

**Authors:** Naoko Hikita, Megumi Haruna, Masayo Matsuzaki, Emi Sasagawa, Minoru Murata, Otgontogoo Oidovsuren, Ariunaa Yura

**Affiliations:** 1 Department of Midwifery and Women’s Health, Division of Health Sciences and Nursing, Graduate School of Medicine, The University of Tokyo, Tokyo, Japan; 2 Mito Saiseikai General Hospital, Ibaraki, Japan; 3 Pharmaceutical Department, Mongolian National University of Medical Sciences, Darkhan-Uul Medical School, Darkhan-Uul, Mongolia; 4 Darkhan-Uul General Hospital, Darkhan-Uul, Mongolia

## Abstract

This cross-sectional study investigated the prevalence of smoking and secondhand smoking (SHS) among pregnant women in Darkhan-Uul Province, Mongolia, using urinary cotinine (UC) levels, and clarified the factors related to SHS exposure. It targeted pregnant women who underwent antenatal health check-ups from November 2015 to January 2016. Self-administered questionnaires and urine samples were used to collect data. Using UC levels as the criterion, it was found that the prevalence of smokers (>100 ng/ml) among 493 pregnant women was 11.8%, while SHS exposure (≥5 ng/ml) among nonsmokers was 44.8%. Older and highly educated women had lower odds of SHS exposure (*p* = 0.006 and 0.002, respectively). Furthermore, nonsmoking pregnant women from homes where smoking was permitted had higher odds of SHS exposure compared to women from homes where smoking was not permitted. These results suggest that community guidance programs, such as home smoking cessation that include families, are necessary.

## Introduction

Secondhand smoke (SHS), also called environmental tobacco smoke, passive smoking, or involuntary smoking, is known to be associated with many adverse health effects on the cardiovascular system and respiratory system^1^, and it increases the risk of sudden infant death syndrome in children^1^. In particular, exposure to SHS during pregnancy harms both the mother and fetus such that it decreases infants’ birthweight^2,3^ and increases the risk of fetal congenital malformation^4^, stillbirth^4^, premature birth^5,6^, stunted growth for gestational age^7^, and pre-eclampsia^8^. Furthermore, prenatal SHS exposure may also increase the risk of asthma^9^, cancer^1^, and impaired neurodevelopment^10–12^ in children.

In Mongolia, approximately 47.6% of females are exposed to SHS at home^13^, which is a much higher percentage than the global average of 35% among women^14^. The high smoking prevalence among male adults (46.3–48.0%)^13,15^ could be a reason for this increased prevalence of SHS exposure among Mongolian women.

There is a large body of research on the risk factors of SHS during pregnancy. In a survey of pregnant women in New York City, race/ethnicity and nativity were found to be predictors of SHS exposure during pregnancy, while lower educational attainment was associated with it^16^. Another study in California revealed that the number of smokers in a household was correlated with SHS^17^, while a study in Spain reported that pregnant women who were younger, of a lower social class and education level, and had a body mass index (BMI) of less than 18.5 were more likely to be exposed to SHS^18^. Younger age and lower education level were identified as risk factors in Malaysia, along with a lower household income^19^. In a study in China, the risk of SHS exposure was greater among pregnant women living in rural areas, who had husbands that smoked heavily, less knowledge about SHS, and no “no smoking” home rule^20^. A South Korean study showed that women with lower gestational age, educational attainment, and those who lived with smoking spouses had higher risk of SHS exposure^21^. However, there have been no studies on SHS targeting pregnant women in Mongolia.

While active smoking is voluntary, passive smoking is involuntary, and even people who are generally cautious of their health may suffer harm if exposed to SHS. SHS exposure during pregnancy can influence both maternal and fetal health. It is important to understand the precise risk factors of SHS among Mongolian pregnant women, as such findings might help devise policies aimed at reducing SHS in the environment surrounding pregnant women and reducing passive smoking. By decreasing SHS exposure among pregnant women, the health of the next generation of Mongolian children could be improved.

The aims of this study are to (1) investigate the current prevalence of smoking and SHS among pregnant women in Mongolia using self-report and urinary cotinine (UC) levels and (2) clarify the factors related to UC-determined SHS.

## Materials and Methods

### Study design and population

This was a cross-sectional study conducted in Darkhan-Uul Province, Mongolia. There are ten public health facilities in Darkhan-Uul Province, including five health centers (i.e., primary health care facilities), three *Soum* hospitals (also primary health care facilities), one general hospital, and one health agency. We conducted the survey in all 10 facilities. The participant recruitment and data collection took place from November 2015 to January 2016.

All pregnant women who visited public health facilities for antenatal health check-ups in Darkhan-Uul Province, Mongolia, and who were considered to be within 20 weeks of conception (calculated from their last menstrual period), were selected as participants for this study. We excluded pregnant women who could not understand Mongolian or had difficulty participating in the study.

The study was performed by trained medical personnel and data were collected using self-administered questionnaires and analysis of urine samples. The staff helped only if it was needed. Participants received ₮3,000 (approximately US $1.50) after the survey as compensation for their transportation expenses.

This study was carried out as a baseline survey of “The Health Improvement Project for Life-style Related Diseases in Darkhan-Uul Aimag, Mongolia,” conducted by the Mito Saiseikai General Hospital with support from the Japan International Cooperation Agency (JICA) Partnership Program. This study was approved by the Research Ethics Committee of the Graduate School of Medicine, The University of Tokyo, Japan (No. 10934) and the Ethical Review Board of the Ministry of Health, Mongolia (No. 06, November 19, 2015). Participation was emphasized as voluntary, and written informed consent was obtained from all the participants. This study’s protocol complied with the principles of the Declaration of Helsinki^22^. All data were analyzed anonymously, and participants’ other identifying information was removed before analysis.

### Questionnaire

The sociodemographic data collected included age, marital status, educational attainment, employment status, monthly household income, number of family members, number of children, nomadic status, and alcohol consumption. Information about the type of dwelling (detached house, condominium, *ger* [traditional tent-like home], or other) and gestational age from the last menstrual period as of the survey (as a reconfirmation) was also obtained.

Participants were asked their current smoking status (including *daily*, *less than daily*, *not at all*, and *don’t know*), self-awareness of SHS exposure (using the question, “How much SHS do you think you are exposed to?” with response options of *not at all*, *a little*, *a lot*, and *very much*), permitted smoking area at home (*not permitted at home*, *permitted in a certain area such as the balcony or bathroom*, and *permitted everywhere including the bedroom*), whether they purposely avoid SHS exposure (using the *yes/no* question, “Do you avoid SHS exposure?”), and an item measuring SHS exposure (using the widely used question, “How often does anyone smoke inside your house?” with response options of *never*, *less than once a month*, *monthly*, *weekly*, *daily*, and *don*’*t know*)^23^. Furthermore, we asked participants to provide the name of a familiar smoker and the amount that person smokes, and created a variable of “presence of familiar smoker” (*none*, *one person*, *two persons*, and *three or more people*) based on their answers. If they did not provide an answer to this question, we considered it as “no smoker present.” Finally, we asked participants about their knowledge related to smoking and SHS using 14 questions.

### Biochemical verification of smoking status

At the survey, all participants were asked to provide a urine sample. All specimens were stored in a cold environment while the survey was conducted. After completing the work for that day, we collected the specimens from all facilities and divided each into two microtubes, which were then stored at −20° Celsius until analysis.

UC levels were measured using the cotinine enzyme-linked immunosorbent assay (ELISA) kit (Calbiotech Inc., Spring Valley, CA, USA). The lower limit of detection for this test kit was set at 1 ng/ml, while the lower and upper limits of quantification were 5 ng/ml and 100 ng/ml, respectively. All UC measurements were carried out at the GYALS Medical Center, LLC, in Ulaanbaatar. The technician used an automated system called the Dynex DS2 ELISA Processing System (Dynex Technologies, Inc., Chantilly, VA, USA) to assess UC levels. Absorbance was assayed with the Microplate Reader MR-96A (Shenzhen Mindray Bio-Medical Electronics Co., Ltd., Shenzhen, China) at a wavelength of 450 nm. The intra assay coefficient of variation was 15%, while the inter assay was 74%.

We determined the smoking status of women using their UC levels. Participants with UC levels of <5 ng/ml (the lowest detectable level of UC) were classified as “not exposed to SHS,” while those with UC levels of ≥5 ng/ml were classified as “exposed to SHS.” We adopted the cutoff of ≥5 ng/ml because a report by the U.S. Surgeon General concluded that even minor exposure to SHS can pose a health risk^1^. Though the cut-off points for distinguishing active smokers from passive smokers ranged from 31.5–550 ng/ml^24^ in urine, in this study, women whose UC levels exceed 100 ng/ml were considered as “biochemically determined smokers”^25^, because a previous study reported that the cut-off point between pregnant women who were exposed to two or more sources of SHS and active smokers was 106 ng/ml^26^, and other previous studies reported that nonsmokers’ UC levels were always less than 100 ng/ml^27,28^. The upper limit of quantification of the ELISA kits used was 100 ng/ml; thus, we decided to adopt the 100 ng/ml as the cut-off point in this study.

Women who answered “daily” or “less than daily” to the self-report question about current smoking status were considered “self-reported active smokers,” and women who answered “not at all” were considered “self-reported nonsmokers”.

### Statistical analysis

We calculated descriptive statistics for all variables. We also performed a bivariate logistic regression analysis to determine the associations between pregnant women’s characteristics and UC levels, after which we performed a multiple logistic regression analysis while adjusting for those variables with *p* values of <0.1 in the bivariate analysis. Multicollinearity was also evaluated using Spearman’s rank-correlation coefficients; if the coefficients between two variables exceeded 0.5, one of the variables was removed from the multiple logistic regression analysis.

All data were analyzed using IBM SPSS Statistics 24.0 for Windows (IBM Corp., Armonk, NY, USA). Two-tailed *p*-values of <0.05 were considered statistically significant.

## Results

### Participants’ characteristics and prevalence of smoking and SHS exposure

A total of 508 pregnant women participated in this study. Fifteen women answered the questionnaires twice at different health facilities; therefore, we used only the questionnaires that were answered first. As such, 493 women’s data were analyzed.

Table 1 shows the characteristics of participants. Maternal age (mean ± SD) was 27.8 ± 6.0 years, 385 (78.1%) were married, and 264 (53.6%) had graduated from a university. Only 43 (8.7%) were nomads/semi-nomads, and 236 (47.9%) lived in condominiums. The mean of gestational age at recruitment was 13.1 ± 5.0 weeks.

**Table 1:** Characteristics of participants (n = 493).

Maternal age (years)	27.8 ± 6.0	
**Marital status**		
Single (Divorced, Bereaved)	103	(20.9)
Married	385	(78.1)
Missing	5	(1.0)
**Educational attainment**		
Low (≤Lower secondary school)	42	(8.5)
Middle (Upper secondary school)	181	(36.7)
High (≥University)	264	(53.6)
Missing	6	(1.2)
**Employment status**		
Employed	192	(39.0)
Self-employed	71	(14.4)
Nomad	13	(2.6)
Unemployed	210	(42.6)
Missing	7	(1.4)
**Monthly household income** ^**a**^		
≤ ₮400,000	141	(28.6)
₮410,000–800,000	237	(48.1)
≥₮810,000	97	(19.7)
Missing	18	(3.6)
**Nomadic status**		
Sedentary	440	(89.3)
Nomadic/semi-nomadic	43	(8.7)
Missing	10	(2.0)
**Type of dwelling**		
Detached house	140	(28.4)
Condominium	236	(47.9)
Ger	92	(18.7)
Others	20	(4.0)
Missing	5	(1.0)
Number of family members^b^	3.7 ± 1.3	
Number of children living together^c^	1.4 ± 1.1	
Gestational age at recruitement^d^	13.1 ± 5.0	

Data are shown as mean ± standard deviation or n (%). ^a^₮20,000 ≒ US$10. ^b^Missing for 21 participants. ^c^Missing for 49 participants. ^d^Missing for 1 participant.

Table 2 shows pregnant women’s current self-reported smoking status and UC levels. Of all pregnant women, 4 (0.8%) reported that they smoked daily, 23 (4.7%) reported that they smoked less than daily, and 438 (88.8%) were nonsmokers; additionally, data on 28 (5.7%) were missing. There were 58 (11.8%) biochemically determined smokers (UC levels >100 ng/ml). By considering only biochemically determined smokers as actual smokers, the remaining pregnant women (435, 88.2%) were UC-determined nonsmokers. Of these, 195 (44.8%) were exposed to SHS.

**Table 2:** Pregnant women’s current self-reported smoking status and urinary cotinine levels (n = 493).

	All		Urinary cotinine concentration					
			<5 ng/ml		5–100 ng/ml		>100 ng/ml	
			Not exposed		SHS exposure		Biochemically determined smokers	
			(n = 240, 48.7%)		(n = 195, 39.5%)		(n = 58, 11.8%)	
**Self-reported smoking status**								
Daily smoker	4	(0.8)	0	(0.0)	0	(0.0)	4	(100.0)
Nondaily smoker	23	(4.7)	2	(8.7)	5	(21.7)	16	(69.6)
Nonsmoker	438	(88.8)	229	(52.2)	175	(40.0)	34	(7.8)
Missing	28	(5.7)	9	(32.1)	15	(53.6)	4	(14.3)

Data are shown as n (%).

### Prevalence of self-reported SHS and factors related to SHS exposure in pregnant women

Table 3 shows the results of smoking and SHS information among 438 self-reported nonsmoking pregnant women. According to their awareness, 267 (61.0%) pregnant women were exposed to SHS a little, a lot, or very much. The result of the widely used SHS exposure measuring question, “How often does anyone smoke inside your house?” showed that 147 (33.6%) pregnant women were exposed to SHS. The mean score of knowledge about smoking among women who were and were not exposed to SHS was not significantly different (*p* = 0.672).

**Table 3:** Smoking and secondhand smoke (SHS) information among self-reported nonsmoking pregnant women (n = 438).

	All	Urinary cotinine concentration			*p* value
		<5 ng/ml Not exposed	5–100 ng/ml SHS exposure	>100 ng/ml Biochemically determined smokers	
**How much SHS do you think you are exposed to?**					0.092
Not at all	158	90 (57.0)	57 (36.1)	11 (6.9)	
A little	206	108 (52.4)	85 (41.3)	13 (6.3)	
A lot	34	12 (35.3)	19 (55.9)	3 (8.8)	
Very much	27	11 (40.7)	11 (40.7)	5 (18.6)	
Missing	13				
**Do you avoid SHS exposure?**					0.040
Yes	334	180 (53.9)	133 (39.8)	21 (6.3)	
No	79	34 (43.0)	34 (43.0)	11 (14.0)	
Missing	25				
**Knowledge about smoking (score)**	9.5 ± 2.9	9.5 ± 3.1	9.5 ± 2.5	9.3 ± 2.6	0.933
**Permitted smoking area at home**					0.001
Not permitted at home	273	162 (59.3)	93 (34.1)	18 (6.6)	
A certain area such as balcony and bathroom	104	40 (38.5)	55 (52.9)	9 (8.6)	
Permitted everywhere including bedroom	30	9 (30.0)	17 (56.7)	4 (13.3)	
Missing	31				
**Women-reported partner’s smoking**					0.174
Not smoking	288	159 (55.2)	110 (38.2)	19 (6.6)	
Smoking	150	70 (46.7)	65 (43.3)	15 (10.0)	
**Presence of familiar smoker**					<0.001
None	202	122 (60.4)	66 (32.7)	14 (6.9)	
One person	198	97 (49.0)	90 (45.4)	11 (5.6)	
Two or more people	38	10 (26.3)	19 (50.0)	9 (23.7)	
**How often does anyone smoke inside your house?**					<0.001
Never	232	146 (62.9)	75 (32.3)	11 (4.8)	
Less than once a month	6	4 (66.6)	1 (16.7)	1 (16.7)	
Monthly	22	10 (45.5)	11 (50.0)	1 (4.5)	
Weekly	35	16 (45.7)	17 (48.6)	2 (5.7)	
Daily	84	28 (33.3)	48 (57.2)	8 (9.5)	
Don’t know	41	15 (36.6)	17 (41.5)	9 (21.9)	
Missing	18				

Data are shown as mean ± standard deviation or n (%). Categorical data was analyzed using chi-squared test, and continuous value was analyzed using one-way analysis of variance. Missing data was excluded from analysis.

Table 4 shows the results of the multiple logistic regression analysis for the factors related to SHS exposure among 404 self-reported nonsmoking pregnant women excluding biochemically determined smokers.

**Table 4:** Multivariate analysis for factors related to SHS exposure (urinary cotinine [UC] ≥5 ng/ml) among self-reported nonsmoking pregnant women excluding biochemically determined smokers (n = 404).

	Crude odds ratio	95% CI^a^	*p* value	Adjusted odds ratio	95% CI^a^	*p* value
**Maternal age (years)**	0.94	(0.90–0.97)	<0.001	0.95	(0.91–0.98)	0.006
**Marital status**						
Single (Divorced, Bereaved)	reference			reference		
Married	0.62	(0.38–1.02)	0.058	0.75	(0.42–1.35)	0.343
**Educational attainment**						
Low ( ≤ lower secondary school)	reference			reference		
Middle (upper secondary school)	0.34	(0.15–0.78)	0.011	0.35	(0.13–0.90)	0.029
High (≥university)	0.25	(0.11–0.57)	0.001	0.24	(0.09–0.60)	0.002
**Permitted smoking area at home**						
Not permitted at home	reference			reference		
A certain area such as balcony and bathroom	2.40	(1.48–3.87)	<0.001	2.25	(1.31–3.88)	0.003
Permitted everywhere including bedroom	3.29	(1.41–7.68)	0.006	2.40	(0.93–6.23)	0.072
**Presence of familiar smoker**						
None	reference			reference		
One person	1.72	(1.13–2.60)	0.011	1.45	(0.89–2.35)	0.138
Two or more people	3.51	(1.54–7.99)	0.003	2.61	(0.98–6.94)	0.055

Multiple logistic regression analysis adjusted for all variables in this table. Missing for 34 responses and 370 responses were used for multiple logistic regression analysis. ^a^Confidence interval.

Older women had lower odds of being exposed to SHS (adjusted odds ratio [AOR] = 0.95, 95% confidence interval [CI]: 0.91–0.98), as did women who had upper secondary school and university education or beyond (AOR = 0.35, 95% CI: 0.13–0.90; AOR = 0.24, 95% CI: 0.09–0.60, respectively). Pregnant women from homes where smoking was permitted in certain areas had twice the odds of SHS exposure compared to women from homes where smoking was not permitted (AOR = 2.25, 95% CI: 1.31–3.88); notably, there was no statistically significant difference in the odds of SHS exposure between women from homes where smoking was not permitted and from homes where smoking was permitted everywhere in the house. Pregnant women who had two or more familiar smokers around them had 2.6 times the odds of SHS exposure than did women without any familiar smokers in their lives, although this was not a significant difference.

## Discussion

This study is the first to examine the current prevalence of active smoking and SHS exposure among pregnant women in Mongolia using a biomarker (UC levels). In this study, 5.5% of pregnant women were smokers based on self-reports, while 11.8% of pregnant women were actual smokers based on UC levels. Furthermore, 61.0% of self-reported nonsmoking pregnant women thought they were exposed to SHS, and according to the widely used SHS measuring question, 33.6% of self-reported pregnant women were exposed to SHS, while 44.8% of UC-determined nonsmoking pregnant women were exposed to SHS (UC levels ≥5 ng/ml). We also identified the factors related to SHS exposure among pregnant women in Mongolia: women of younger ages, who had low educational attainment, and who were from homes where smoking was allowed were more likely to be exposed to SHS.

### Participants’ characteristics

In this study, we recruited almost all the eligible pregnant women who visited public health facilities for antenatal health check-ups, though we could not grasp the actual participation rate. Of these, 90.3% of pregnant women graduated from upper secondary school or higher. In Mongolia, 93.7% of women are enrolled in upper secondary school^29^; thus, we could say our population was representative. Furthermore, Mongolian women’s literacy rate is 97.3%^30^; thus, we assumed that the participants’ comprehension abilities were sufficient to answer the questionnaire.

Only 8.7% of the participants were nomads, although approximately 18% of households live as nomadic shepherds in Mongolia^31^. The population of nomadic shepherds in Darkhan-Uul is 3.7%.

Given these characteristics, we can assert that our participants represented the general population of pregnant women in Darkhan-Uul.

### Prevalence of active smoking and SHS

We used UC levels to assess the prevalence of active smoking and SHS. We found that 11.8% of pregnant women potentially smoked based on UC levels, although the self-reported smoking rate was 5.5%. Furthermore, 44.8% of UC-determined nonsmoking pregnant women were exposed to SHS (UC levels ≥5 ng/ml), although according to the widely used SHS measuring question, 33.6% of self-reported pregnant women were exposed to SHS. A previous study conducted in Mongolia reported that 6.8–6.9% of females smoked^13,15^, while 47.6% of females were exposed to SHS in their homes^13^. Although the prevalence of smoking and passive smoking based on self-reports was somewhat similar to previous studies, the prevalence of UC-determined smokers was different from previous ones. If we considered missing data as smokers, the prevalence of self-reported smokers would not be so different from that of UC-determined smokers, although some smokers lie and say that they are nonsmokers.

We suspect that it is more difficult for women to declare that they are smokers than it is for men because of social pressure or the need to maintain appearances. This is perhaps especially difficult for pregnant women, who must not only think about the health of their fetus, but also about what the public would expect of them as future mothers. Therefore, pregnant smokers might be even less likely than other women to answer honestly, and for that reason, actual smokers were mixed with self-reported nonsmokers, which made it difficult to grasp the prevalence of passive smoking accurately. This suggests that surveys conducted in Mongolia in the past probably provided inaccurate active and passive smoking rates, given that they all used self-report questionnaires. Accordingly, our study might offer a more accurate picture of the prevalence of active and passive smoking among pregnant women in Darkhan-Uul, Mongolia. However, cotinine clearance is noticeably higher during pregnancy, with a half-life of a little less than nine hours^32^, and the measurement of UC levels is not the gold standard, especially when we want to verify passive smoking; thus, we should understand its limitation.

### Factors related to SHS exposure

Although this was the first study to examine the factors influencing SHS exposure among pregnant women in Mongolia, we did not identify any novel factors related to SHS exposure specific to developing countries. Women of a younger age were more likely to be exposed to SHS in this study, which supports the findings of several previous studies^18,19,33^.

We also found that women with low education levels had higher odds of SHS exposure. This finding coincides with the findings of previous studies^16,18,19,21,33^. Importantly, knowledge of smoking and SHS was not associated with SHS exposure, suggesting that even if women have the knowledge, they might not avoid SHS. This finding contradicts that of a previous study^20^. One possible reason for the contradiction is that the questions assessing smoking and SHS knowledge that we used were not sensitive enough to measure participants’ knowledge of smoking or SHS. Another possible reason is that the knowledge and cooperation of the surrounding people, not just the knowledge and effort of pregnant women, has a great influence on SHS exposure and can prevent passive smoking.

Women from homes where smoking was allowed had twofold greater odds of SHS exposure compared to women from homes where smoking was not allowed. While this result supports that of a previous study^20^, we curiously did not find a difference between women from homes where smoking was not allowed and those from homes where smoking was allowed everywhere in the house, even after adjusting for certain variables. As we expected that women from homes where smoking was allowed everywhere in the house would be at greatest risk of SHS, this result was surprising. One reason may be that the sample size might have been too small and the power was not enough to reveal a significant difference.

Notably, the number of familiar smokers was not related to SHS exposure in our study, which contradicts the results of previous research^17^. This inconsistency might be because we had participants list the specific familiar smokers and counted the number from the names listed; some pregnant women might not have reported these data accurately because they found it too troublesome. Alternatively, tobacco smoke can easily pass through non-airtight physical barriers—thus, even if nobody smokes around them, they might still find it difficult to avoid the smoke completely.

Though cotinine is a sensitive method of assessing active and passive smoking, if it is not available, self-reported SHS exposure might provide a good estimation of such exposure if certain information – such as age, education level, and whether smoking is permitted at home – is obtained along with self-reported exposure^34^. Given this, health professionals would be able to assess SHS exposure among pregnant women.

### Strengths and limitations

Above all, this is the first study to have measured pregnant women’s active smoking and SHS exposure using a biological marker in Mongolia. Assessing SHS exposure using biological markers is rare in middle-income countries because of some difficulties, such as the lack of funds, measurement instruments, and insufficient infrastructure. For this reason, our results are particularly valuable.

Despite its strengths, the study has several limitations as well. First, we conducted this research for only two months during winter. Outside temperature differs substantially between summer and winter in Mongolia, which means that the timing of data collection might have influenced the occasion or frequency of SHS exposure among pregnant women. A previous study reported that UC levels in winter were higher than in summer^18^ because people smoke indoors with poorer ventilation to avoid the cold weather. Thus, we need to conduct a survey covering the full range of seasons to clarify the prevalence of SHS exposure among pregnant women in Mongolia. Nevertheless, we recruited pregnant women who visited public health facilities within 20 weeks of conception, and most women who attended during the study period participated in our study. In fact, the participants’ characteristics were representative of Mongolian woman. Therefore, our results can be generalized to pregnant women in Darkhan-Uul Province, at least for the winter.

Second, the inter-plate assay %CV was fairly high, indicating that the results must be cautiously interpreted. However, since we used UC concentration as categorical data, some gaps resulting from the analysis were likely covered. Third, although our sample was representative, the results cannot be generalized to the entire Mongolian population because lifestyles differ substantially throughout the country. However, as Darkhan-Uul is the third largest populated province in Mongolia and most of the participants lived in urban areas, it is possible to generalize the results to other urban areas, such as the capital city, Ulaanbaatar. Fourth, we could only collect UC data once during pregnancy, which means that we cannot conclude that women were exposed to the same amount of SHS throughout their pregnancy. However, a previous study reported that as gestational age increases, pregnant women are exposed less to SHS^21^. Therefore, the prevalence of SHS exposure of pregnant women in the second or third trimester is not likely to have exceeded that found in our study. Fifth, the sample size might have been too small, resulting in insufficient power to reveal some significant differences. To uncover the risk factors for SHS exposure that could not be determined in this study, future research should include a larger sample size.

Regarding the clinical implications of this study, our results suggest that some pregnant women are unwilling to declare that they are current smokers, which leads to underreporting of smoking rates. As such, health professionals would be wise to provide education on the harm caused by smoking and SHS exposure during pregnancy to pregnant women who are self-reported smokers, as well as to those who are self-reported nonsmokers. Specifically, we found that pregnant women who were younger, had low educational attainment, and permitted smoking at home were at a higher risk of exposure to SHS; therefore, health professionals need to understand this information while providing education.

## Conclusion

Our study determined the prevalence of active and passive smoking among pregnant women in Darkhan-Uul Province, Mongolia, using self-report and UC levels. Furthermore, we identified the factors influencing UC-determined SHS exposure. Especially for pregnant women, honestly reporting that they are active smokers might be difficult because of factors pressuring people to avoid smoking during pregnancy, such as social pressure, a sense of guilt, or worrying about appearances. Therefore, health professionals must be careful when obtaining self-reported smoking status. Furthermore, health education to reduce SHS exposure during pregnancy, such as a program on home smoking cessation, should be provided to the community, including family members, because the knowledge and effort of pregnant women alone cannot prevent passive smoking. Future research should focus on modifiable risk factors for SHS exposure during pregnancy and develop corresponding prevention strategies.
